# Young Adults with Anxiety Disorders Show Reduced Inhibition in the Dorsolateral Prefrontal Cortex at Higher Trait Anxiety Levels: A TMS-EEG Study

**DOI:** 10.1155/2024/2758522

**Published:** 2024-05-30

**Authors:** Lena Pokorny, Lea Biermann, Eva Breitinger, Tomasz Antoni Jarczok, Daniel Wagner, Jasper Vöckel, Stephan Bender

**Affiliations:** ^1^Department of Child and Adolescent Psychiatry, Psychosomatics, And Psychotherapy, University of Cologne, Faculty of Medicine and University Hospital Cologne, Cologne, Germany; ^2^Department of Child and Adolescent Psychiatry and Psychotherapy, KJF Klinik Josefinum, Kapellenstrasse 30 86154, Augsburg, Germany; ^3^Department of Psychiatry and Psychotherapy, University of Cologne, Faculty of Medicine and University Hospital Cologne, Cologne, Germany

## Abstract

**Background:**

The neuropathology of anxiety disorders, including specific phobias, social phobias, and generalized anxiety disorders (GAD), has been believed to be rooted in a reduced inhibition of limbic areas by the dorsolateral prefrontal cortex (DLPFC). Trait anxiety has been linked to insufficient recruitment of DLPFC mechanisms for attentional control. Despite limited research on individuals with anxiety disorders, our study utilized transcranial magnetic stimulation to assess DLPFC cortical activity and emotional states using the N100 as an indicator of GABA-B-mediated cortical inhibition. Additionally, we aimed to correlate trait anxiety scores with cortical activity.

**Methods:**

A total of 20 subjects with social phobia and GAD and 21 subjects with specific phobia were compared to 24 control subjects regarding their inhibitory N100 in the DLPFC. Therefore, TMS was applied on the left and right DLPFC during an emotional task with fearful, angry, and neutral faces and a rest condition.

**Results:**

Smaller N100 amplitudes after DLPFC stimulation were found in subjects with social phobia, GAD, and social phobias compared to the control group. Furthermore, a correlation between trait anxiety scores and smaller N100 amplitudes, independent of group effects, was found.

**Conclusion:**

There appears to be a decrease in GABA-B-mediated cortical inhibition in the DLPFC in subjects with anxiety disorders. The correlation between trait anxiety and N100 amplitudes suggests a trait-related modulation of cortical inhibition.

## 1. Introduction

Cognitive control processes that enable goal-directed behavior are necessary in daily life. They are responsible for shielding attention from distracting or irrelevant information [1]. One brain region associated with cognitive control and attention is the dorsolateral prefrontal cortex (DLPFC) [2].

The DLPFC has been thought to exert its controlling influences during emotional activation through top-down inhibition of the amygdala [3, 4]. For this reason, it has been suggested to play an important role in fear processing. Neuroimaging studies have shown that activation in the prefrontal cortex decreases with increasing levels of anxiety [5]. A close connection between prefrontal areas and the amygdala with top-down and bottom-up communication pathways has also been described [6]. The described prefrontal top-down regulation of the amygdala and limbic system has been suggested to be mediated by GABAergic interneurons [1, 7, 8]. Inhibitory interneurons are thought to be critical in shielding a behavioral target from the influences of interfering stimuli [1]. Thus, a deficit in prefrontal inhibition could contribute to ineffective top-down control and, thus, less efficient shielding. This could impair the ability to maintain goal-directed behavior in the context of fear-related stimuli.

Because of the involvement of the DLPFC in the processing of fear, it is reasonable to consider the DLPFC in the context of the pathophysiological mechanisms of anxiety disorders. Anxiety disorders such as specific phobias, social phobias, or generalized anxiety disorders (GAD) have the highest lifetime prevalence among mental disorders [9, 10] and are often diagnosed already in childhood or adolescence [11, 12].

Impairments in emotion regulation and top-down attentional control have been found in patients with social phobia and GAD [13]. It has been described that common deficits in both anxiety disorder groups may be related to a more general difficulty in recruiting regions involved in top-down attentional control.

Cortical processes and, thus, pathophysiological mechanisms can be investigated using a combination of transcranial magnetic stimulation (TMS) and electroencephalography (EEG) [14]. TMS-evoked potentials (TEP) are time-locked neuronal responses to TMS pulses. A frequently investigated TEP is the so-called N100, which has been described to reflect metabotropic gamma-aminobutyric acid (GABA) B receptor-mediated inhibitory processes in both the motor cortex (M1) and DLPFC [15–18]. GABAB receptors are thought to be highly concentrated in the basolateral amygdala [19]. For this reason, it has been discussed that GABAB receptors play an important role in the pathophysiology of anxiety disorders [20–22]. The N100 therefore offers the possibility of a noninvasive investigation of GABAB receptor-mediated inhibitory processes in the DLPFC.

In a previous study, we found a reduced prefrontal top-down inhibition in young adults with specific phobias compared to a control group [23, 24]. We have argued that such an inhibitory deficit in top-down regulatory processes in subjects with specific phobias may be due to higher levels of trait anxiety. In the literature, trait anxiety has increasingly been associated with insufficient recruitment of DLPFC mechanisms involved in attentional control [3, 25, 26]. Furthermore, the severity of anxiety symptoms has been found to be correlated with the level of trait anxiety [27]. Since subjects with anxiety disorders, especially with GAD and social phobias, show high levels of trait anxiety [28], the question remains whether such an inhibition deficit can also be found in these more severe groups of anxiety disorders and whether it is more pronounced than in specific phobia.

In light of these considerations, we assessed the N100 amplitude at rest and during an emotional n-back task. We expected a reduced inhibition level, as reflected in a smaller N100 amplitude, at rest in anxious subjects compared to controls. We combined social phobia and GAD—both presumably with higher trait anxiety scores and higher severity of anxiety symptoms—into one group and compared them with a group with specific phobia—presumably with slightly lower trait anxiety scores—and a control group. In addition, we expected an enhanced inhibitory deficit by emotional processing during the emotional n-back task since cortical activity can be modulated by the processing of emotions [29]. Since anxiety-inducing stimuli can also affect DLPFC functioning, it is possible that the inhibitory ability of DLPFC is influenced by the processing of fearful facial expressions [29–32]. This would be reflected in smaller N100 amplitudes in response to fearful compared to neutral facial expressions.

Secondly, we expect that smaller N100 amplitudes would correlate with greater trait anxiety.

## 2. Methods and Material

The methods and material were described similarly to the methods and material section from Pokorny et al. [23, 24], as the same experimental procedure was applied to an additional group with a different anxiety disorder.

### 2.1. Subjects

Sixty-eight subjects between 18 and 25 years old participated in the study. Twenty subjects were diagnosed with a social phobia, or GAD; 22 subjects had a specific phobia; and 26 subjects did not have any psychiatric disorder and represented the control group. Two subjects of the control group had to be excluded from statistical analyses due to unremovable prolonged TMS (“decay”) artefacts [33]. Due to a technical problem with the presentation software, two additional subjects, one from the specific phobia group and one from the control group, were excluded from the n-back task analyses. The diagnoses of the different anxiety disorders and comorbid mental disorders were assessed using the Structured Clinical Interview (SCID) based on the research criteria of the fifth edition of the Diagnostic and Statistical Manual of Mental Disorders (DSM-5) [34, 35].

Depression symptoms were measured considering the Hamilton Depression Rating Scale [36] according to the structured interview by Williams [37]. There were no other comorbid mental or neurological disorders. In the group of specific phobia, fifteen subjects had a specific phobia of an animal type, one subject of blood-injection injury, three subjects of the natural environment, and three subjects of situational type. Detailed sample characteristics of samples can be found in Table 1. All subjects were free of psychotropic substances. Subjects were screened for TMS exclusion criteria according to Rossi et al. [38]. The trait anxiety was assessed by the state-trait inventory (STAI) [39].

### 2.2. Transcranial Magnetic Stimulation (TMS)

Single-pulse biphasic TMS was applied via a MagPro ×100 with a MagOption stimulator and a 75 mm outer diameter figure-of-eight coil (MCF-B65, Magventure, Farum, Denmark). The coil was held over electrodes F5 and F6 by a trained examiner. For TMS, electrode positions F5/F6 were used, as this has been shown to be a clinically useful method to localise the DLPFC when individual structural MRI data are not available [40, 41]. TMS pulses were applied by Presentation Software 18.1 (NeuroBehavioral Systems, Berkley, USA).

To measure motor evoked potentials (MEPs), an electromyogram (EMG) was applied with an electrode attached to the first dorsal interosseous muscle and a reference electrode attached to the proximal phalanx of the index finger of the right hand. Resting motor threshold (RMT) was determined by threshold hunting ([42] MTAT 2.0; available online at https://www.clinicalresearcher.org/software.htm). The stimulation intensity for TMS was 120% of the individual RMT. A TMS protocol with a total of 90 stimuli with an interstimulus interval of 5 to 8 s (mean 6.5 s) was applied to the left and right DLPFC in the resting condition (45 pulses left, 45 pulses right).

### 2.3. 1-Back Task

195 standardised and evaluated emotional facial expressions of different actors were presented in our 1-back paradigm in a pseudorandomised order using Presentation Software 18.1.

**Figure 1:**
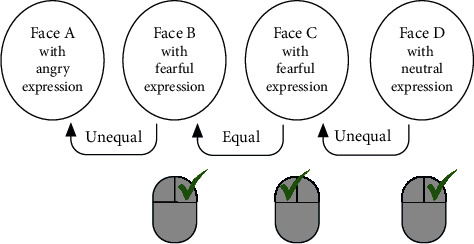
Exemplary visual illustration of the 1-back task from left to right. The stimuli consisted of different faces of different people. These faces were divided into three different groups of facial expressions: fearful, angry, and neutral facial expression. The subjects had to decide by remembering the facial expression whether a facial expression was the same (left mouse click) or different (right mouse click) to the previously shown group of facial expressions (example above: angry ≠ fearful = fearful ≠ neutral).

The subjects were required to remember the emotional expression (fearful, angry, and neutral) and decide by mouse click whether the emotion presented matched the emotion previously presented by another actor (1-back task, Figure 1). This task design activates the working memory and thereby the DLPFC [43, 44].

For our investigation of the interference of emotional processing and everyday cognitive control, an emotional 1-back task with a low load on working memory was sufficient and appropriate to ensure that the emotional expression was processed. The trials were divided into three blocks to prevent exhaustion. Each trial lasted 2.5 s, of which the emotional facial expression was shown for 1 s, followed by 1.5 s of blank screen with a fixation cross. During the blank screen, the subjects could give a response while simultaneously retaining the emotion in their working memory for the next trial. Since the processing of visual n-back tasks occurs predominantly in the right hemisphere [43], TMS was applied to the right DLPFC. During the 1-back task, 20 TMS stimuli on average were applied to the right DLPFC after fearful, 20 after angry, and 20 after neutral emotional stimuli. Unfortunately, one stimulus from one stimulus category was not presented in each protocol, so a total of 59 instead of 60 TMS stimuli were applied. The TMS pulses were applied 250 ms after the offset of the emotional facial expression, on average every 3.4 trials. The test phase was always preceded by a short training session.

The order of the 1-back task and TMS at rest was counterbalanced across subjects.

### 2.4. Electroencephalographic Recordings

We used 64-channel BrainCaps in an equidistant montage with sintered Ag/Ag-Cl electrodes (Easycap GmbH, Herrsching, Germany). All impedances were kept at <5 k*Ω*. EEG channels were named according to the nearest corresponding channel in the 10-20 system [45]. During recording, Cz served as a reference electrode. 1 cm below the left eye and right eye, an infraorbital electrode was placed. We recorded the continuous DC EEG using the BrainVision recorder (Brain Products GmbH, Gilching, Germany) at a sampling rate of 5000 Hz.

### 2.5. EEG Data Analysis

#### 2.5.1. Signal Processing

The BrainVision Analyzer (BrainProducts GmbH, Gilching, Germany) was used for offline processing of the EEG data. After reducing the sampling rate to 500 Hz, an average reference was used for rereferencing. To examine the TMS-triggered N100 at rest and during the 1-back task, the EEG data were segmented into intervals of 1 s (0.5 s before the TMS pulse and 0.5 s after). Interpolation of the time window between 10 ms and 20 ms around the TMS pulse was performed to avoid contamination of the EEG segments by the TMS pulse artefact. Trials with muscle artefacts, movements, and electrode artefacts were removed, accounting for 1.55% of the trials. In order to avoid contamination of the baseline due to possible broadening of the TMS pulse artefact by the downsampling procedure, the time window from -110 ms to -10 ms before the TMS stimulus was used as a baseline. To remove artefacts such as eye blinks and movements, independent component analysis was applied. To correct for any drifts, a DC trend correction was applied [46], although data inspection did not reveal any systematic influences of DC detrending. All trials were finally averaged for each condition and stimulation side. For the resting condition, the 45 pulses on each side (F5, F6) were included in the average. For the task, 59 pulses were included in the average (19 or 20 stimuli per emotion).

#### 2.5.2. N100 DLPFC Analysis

The highest negative peak at ipsilateral F5 after left TMS or F6 after right TMS in the interval 80-140 ms was determined as TMS-evoked N100. The time window and electrodes of interest were selected according to previous literature on TMS-EEG [16, 47]. Around the peak, mean amplitudes of -10 to 10 ms were exported.

### 2.6. Statistics

Statistical analyses were performed using IBM SPSS Statistics 29 software (IBM Corp., Armonk, NY, USA; Version 29).

A 2 × 3 ANOVA with repeated measures with the within-subject factor STIMULATION SIDE (TMS to the DLPFC on the left and right side) and the between-subject factor GROUP (social phobia and GADs, specific phobias, and control group) was performed to compare the N100 amplitudes at rest after TMS to the DLPFC. To adjust for possible influences of depressive symptoms, an ANCOVA was calculated with the factors STIMULATION SIDE and GROUP and the covariate DEPRESSIVE SYMPTOMS measured by the Hamilton Depression Rating Scale.

The 1-back task effect on N100 amplitudes was analyzed by a 2 × 3 ANOVA with the within-subject factor CONDITION (rest vs. 1-back task) and the between-subject factor GROUP (social phobia and GADs, specific phobia, and control group).

The effect of the emotional facial expressions on TMS-evoked N100 amplitudes was tested by a 3 × 3 ANOVA with the within-subject factor EMOTION (fearful, angry, and neutral faces) and the between-subject factor GROUP (social phobia and GADs, specific phobia, and control group).

To investigate the possible connection between trait anxiety and the N100 amplitude, a linear regression using the trait anxiety score as a predictor of the N100 amplitude on the left and right stimulation sides after TMS of the DLPFC at rest was calculated. Furthermore, an ANCOVA with the N100 over both sides as a covariate, trait anxiety scores as a dependent variable, and the three groups as an independent variable was calculated to investigate whether the three groups differ in trait anxiety when the influence of the N100 is removed.

Significant results were assumed at a *p* value< 0.05. Significant interactions were followed up by one-way ANOVAs and post hoc tests. When necessary, values were given in the Greenhouse-Geisser and Bonferroni-Holm corrected forms.

## 3. Results

### 3.1. TMS-Evoked N100 at Rest

**Figure 2:**
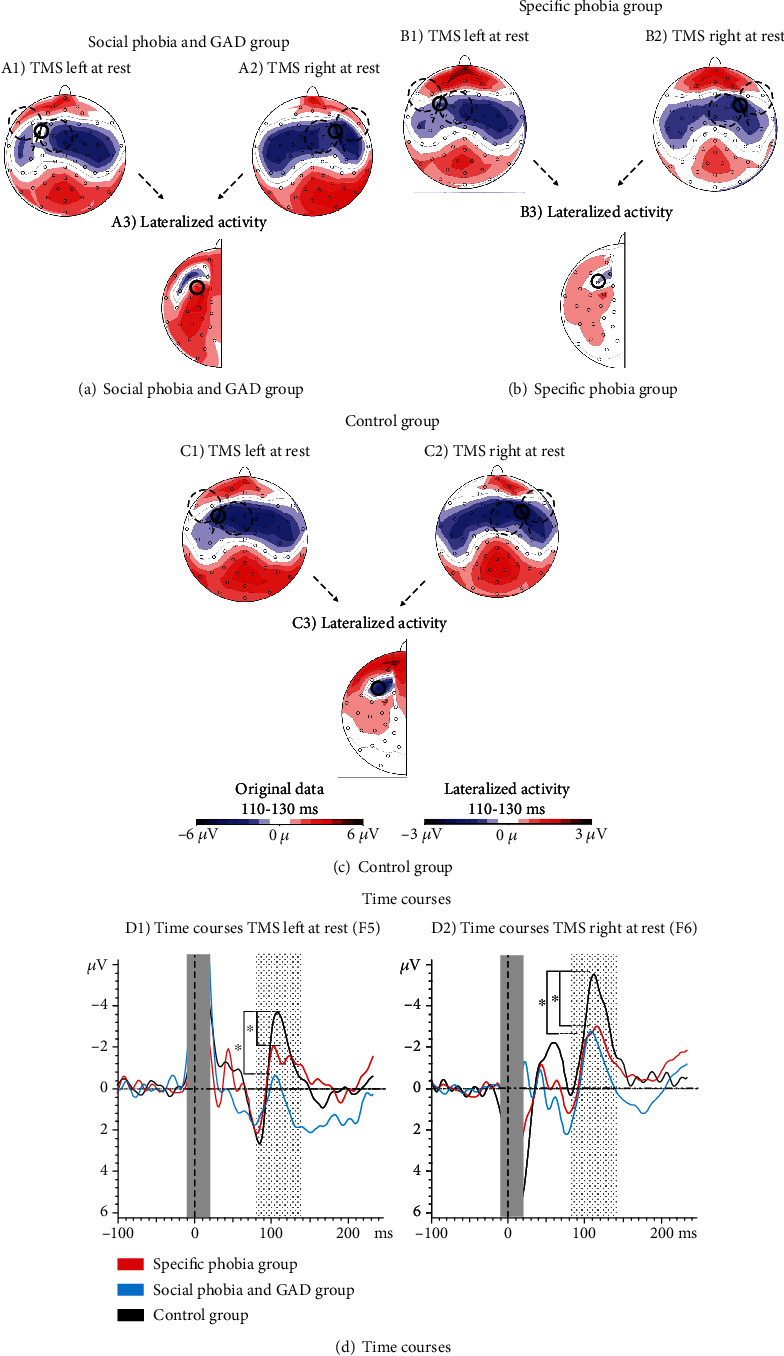
Topographic distributions and time courses of the three groups for left- and right-sided TMS. ^∗^ indicates significant differences in the time courses.

The 2 × 3 ANOVA showed a significant main effect of GROUP (*F*(2, 63) = 3.39, ⁣^∗^*p* = 0.020, partial *η*^2^ = 0.10). The groups with social phobia and GAD and with specific phobias showed smaller N100 amplitudes over both stimulation sites compared to the control group but did not differ significantly from each other (social phobia and GAD vs. controls: *t*(42) = 2.30, ⁣^∗^*p* = 0.039, *d* = 0.70; specific phobia vs. controls: *t*(44) = 2.10, ⁣^∗^*p* = 0.042, *d* = 0.62; social phobia and GAD vs. specific phobia: *t*(40) = 0.33, *p* = 0.372, *d* = 0.10; Figure 2). All N100 amplitudes with standard deviations and latencies are shown in Table 1 in the supplementary material.

**Figure 3:**
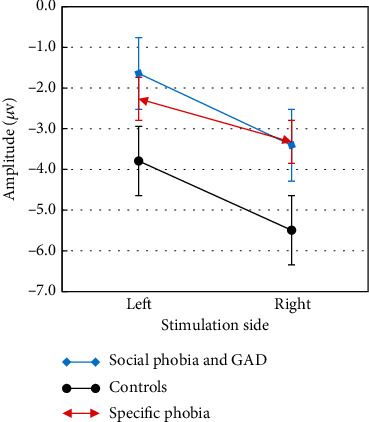
N100 amplitudes in *μ*V of the three analyzed groups: social phobia and generalized anxiety disorder (GAD) group, specific phobia group, and the control group on both stimulation sides with standard error bars.

Furthermore, right sided stimulation evoked larger N100 amplitudes irrespective of the group (main effect SIDE: *F*(1, 63) = 7.87, ⁣^∗∗^*p* = 0.007, partial *η*^2^ = 0.11; Figure 3).

There was no significant interaction between STIMULATION SIDE and GROUP (*F*(2, 63) = 0.18, *p* = 0.839, partial *η*^2^ = 0.01).

After adjusting for depressive symptoms, the N100 still differed significantly between the three groups (main effect GROUP: *F*(2, 62) = 2.41, ⁣^∗^*p* = 0.049, partial *η*^2^ = 0.07).

### 3.2. TMS-Evoked N100: 1-Back Task

**Figure 4:**
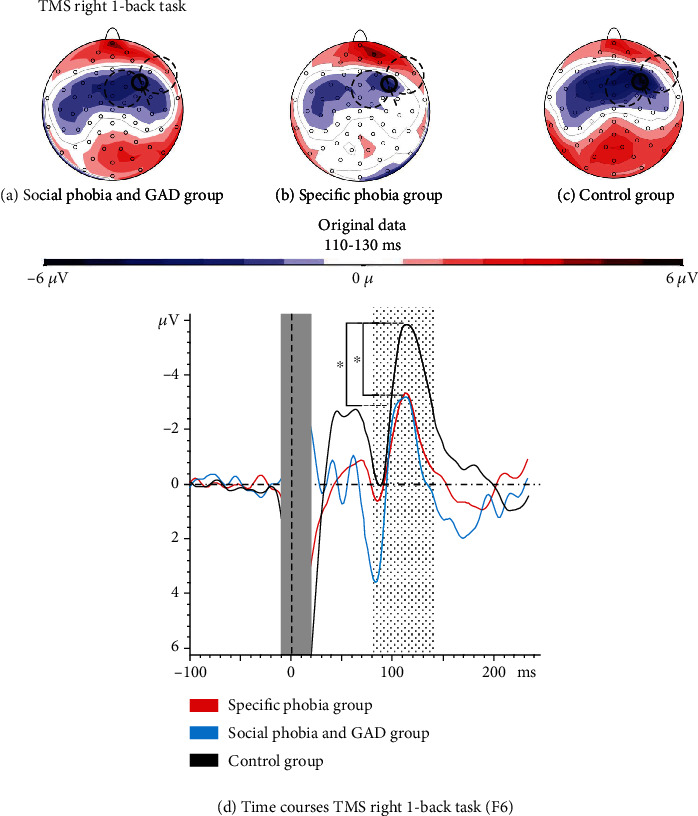
Topographic distributions and time courses of the three groups for right-sided TMS during the 1-back task. ^∗^ indicates significant differences in the time courses.

There were no significant changes in the N100 amplitudes by working memory processing during the 1-back task (CONDITION: *F*(1, 61) = 0.53, *p* = 0.467, partial *η*^2^ = 0.01), and there was no interaction between the 1-back task and the three diagnostic groups (CONDITION × GROUP*F*(2, 61) = 0.87, *p* = 0.423, partial *η*^2^ = 0.03). Nevertheless, the main effect of GROUP remained significant during the 1-back task (*F*(2, 61) = 5.14, ⁣^∗∗^*p* = 0.009, partial *η*^2^ = 0.14; Figure 4). The group with social phobia and GAD and specific phobias showed again smaller amplitudes compared to the control group (social phobia and GAD vs. controls: *t*(41) = −2.68, ⁣^∗^*p* = 0.015, *d* = −0.82; specific phobia vs. controls: *t*(42) = 2.47, ⁣^∗^*p* = 0.018, *d* = −0.75), but did not differ among themselves (social phobia and GAD vs. specific phobia: *t*(39) = 0.34, *p* = 0.369, *d* = 0.11; Figure 4).

### 3.3. TMS-Evoked N100: Effect of Emotional Facial Expression

**Figure 5:**
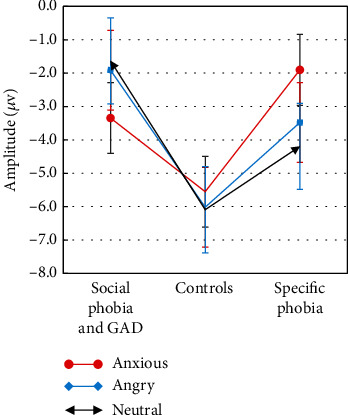
N100 amplitudes in *μ*V of the three analyzed groups: social phobia and generalized anxiety disorder (GAD) group, specific phobia group, and the control group, divided according to the three emotional facial expressions (anxious, angry, and neutral) with standard error bars.

There was no main effect of EMOTION (*F*(1.7, 100.9) = 2.71, *p* = 0.748, partial *η*^2^ = 0.004), but a significant interaction EMOTION × GROUP (*F*(3.3, 100.9) = 2.37, ⁣^∗^*p* = 0.035, partial *η*^2^ = 0.07; Figure 5).

**Figure 6:**
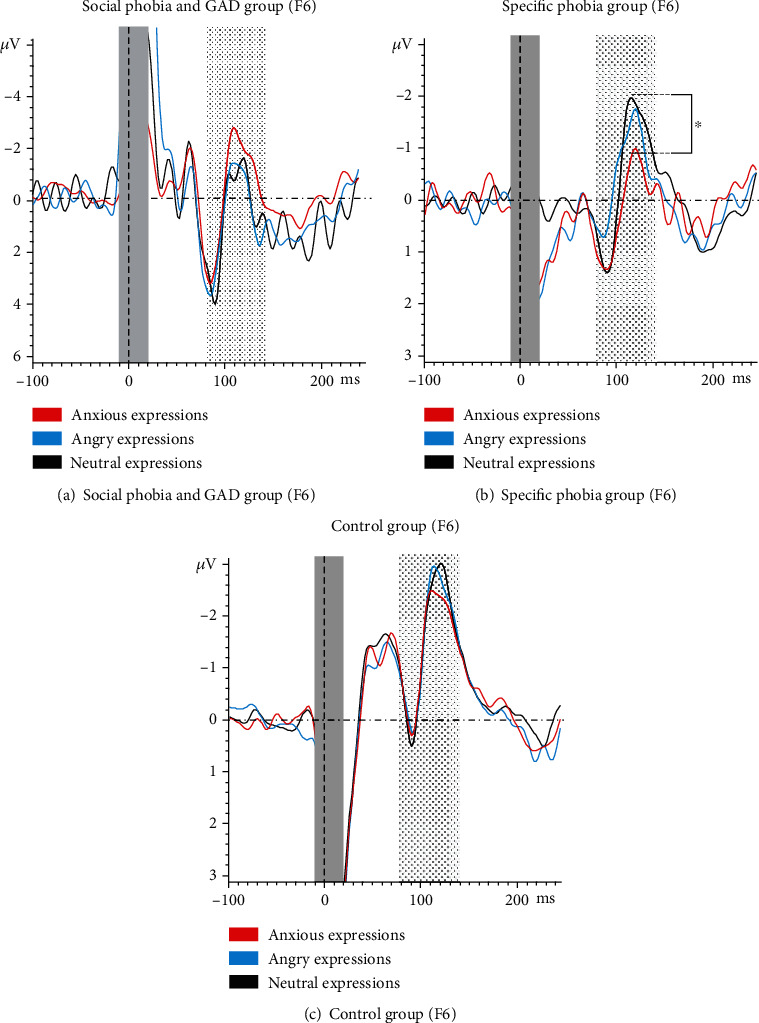
Time courses of the three groups, divided according to the three emotional facial expressions (anxious, angry, and neutral). ^∗^ indicates significant differences in the time courses.

Following the interaction, the one-way ANOVA for the social phobia and GAD group and the control group did not show a significant main effect EMOTION (social phobia and GAD: *F*(1.5, 28.9) = 0.96, *p* = 0.373, partial *η*^2^ = 0.05; control group: *F*(2, 44) = 0.34, *p* = 0.713, partial *η*^2^ = 0.02; Figure 6). Only the one-way ANOVA for the specific phobia group showed a significant main effect of EMOTION (*F*(1.5, 30.9) = 4.89, ⁣^∗^*p* = 0.021, partial *η*^2^ = 0.20; Figure 6). In reaction to fearful facial expressions, the specific phobia group showed significantly smaller N100 amplitudes compared to neutral ones (*t*(20) = 3.87, ⁣^∗∗^*p* < 0.001).

### 3.4. Connection between Trait Anxiety and N100

**Figure 7:**
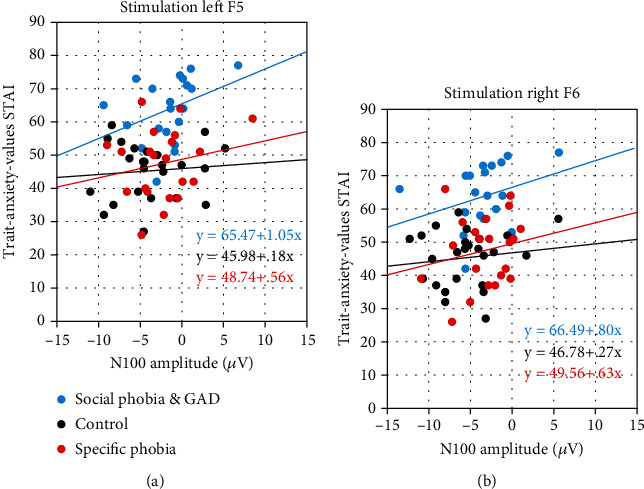
Linear regression with the trait anxiety score as a predictor of the amount of the N100 amplitude on the left (a) and right (b) stimulation side after TMS of the DLPFC at rest. The regression equations are divided according to the three groups: social phobia and generalized anxiety disorder (GAD) group (blue), specific phobia group (red), and the control group (black).

The linear regression using the trait anxiety score as a predictor of the N100 amplitude yielded a significant effect of trait anxiety on the N100 amplitude for both stimulation sides (Figure 7). For the left stimulation side, the trait anxiety score explained about 10% of the N100 amplitude variance (*R*^2^ = 0.08; *F*(1, 63) = 5.11, ⁣^∗^*p* = 0.027). The higher the trait anxiety score, the smaller and thus more positive was the N100 amplitude (0.87 *μ*V per trait anxiety score point) (*b* = 0.87; *t*(63) = 2.26; ⁣^∗^*p* = 0.027). For the right stimulation side, the trait anxiety score explained a slightly smaller amount of N100 amplitude variance (*R*^2^ = 0.06; *F*(1, 63) = 4.10, ⁣^∗^*p* = 0.047). Again, the higher the trait anxiety score, the smaller and thus more positive the N100 amplitude (0.82 *μ*V per trait anxiety score point) (*b* = 0.82; *t*(63) = 2.03; ⁣^∗^*p* = 0.047).

In the next step, we checked whether the correlation between trait anxiety and N100 amplitude mediated the group effect. However, an ANCOVA showed that the three groups still differed significantly regarding their trait anxiety scores after adjusting for the effects of the N100 amplitude (*F*(2, 59) = 13.40, ⁣^∗∗^*p* < 0.001, partial *η*^2^ = 0.31). Post hoc tests showed that trait anxiety scores were significantly higher in the group of social phobia and GAD compared to both the control group and specific phobia group (social phobia and GAD vs. control: ⁣^∗∗^*p* < 0.001, *M*_Diff_ = 16.83, 95% CI [10.81, 22.86]; social phobia and GAD vs. specific phobia: ⁣^∗∗^*p* < 0.001, *M*_Diff_ = 15.66, 95% CI [9.59, 21.73]). The control group did not differ significantly from the specific phobia group (*p* = 0.691, *M*_Diff_ = 1.17, 95% CI [-4.71, 7.06]).

## 4. Discussion

A reduced prefrontal inhibition could be found in young adults with social phobia or GAD compared to a control group under a resting condition. This finding is in line with our previous findings on subjects with specific phobia [23, 24, 48]. This inhibition impairment was reflected in smaller amplitudes of the TMS-evoked N100 after TMS of the DLPFC in the group with anxiety disorders. Descriptively, the group with social phobia and GAD showed an even more pronounced inhibition compared to the group with specific phobia. However, this difference did not gain statistical significance.

The main finding of this paper was a correlation between the trait anxiety scores and the N100 amplitude. We found that the higher the trait anxiety score, the smaller and thus more positive the N100 amplitude. This was true for both stimulation sides. This effect seemed independent of and additional to the group effects on the N100 amplitude.

Furthermore, we replicated that there were no significant changes in the N100 amplitudes by working memory processing during a 1-back task. When emotional processing was examined by memorizing emotional facial expressions, we could not replicate the effect of smaller N100 amplitudes in reaction to fearful compared to neutral facial expressions for the group of social phobia and GAD that we found in the group with specific phobias. There were no significant differences regarding facial emotional expressions between the social phobia and GAD group and the control group.

### 4.1. TMS-Evoked N100 and Trait Anxiety Scores

The N100 in the DLPFC is thought to occur as a lateralized negative deflection at the stimulation site [49, 50] and, as a parameter of intracortical inhibition, likely reflects GABAB reactivity in the DLPFC [17, 51, 52]. We found such an ipsilateral N100 at the stimulation site, at electrode F5 for left-sided stimulation, and at F6 for right-sided stimulation. This N100 showed significantly smaller amplitudes in the social phobia and GAD group compared to the control group which replicates our findings of a smaller N100 in subjects with specific phobia. Descriptively, the N100 was even smaller in the group with social phobia and GAD compared to the specific phobia group. Smaller N100 amplitudes could indicate that subjects with social phobia and GAD also have impaired GABAB-mediated inhibition in the DLPFC.

Importantly, the group of social phobia and GAD was free of any psychotropic drugs at the time of participation, just like the group with specific phobia. Therefore, drug treatment can be excluded as a cause of the N100 reduction. Furthermore, the group difference with reduced prefrontal inhibition in anxiety disorders remained when adjusted for depression scores. This suggests that the comorbid mild depressive episodes were not a decisive factor.

In our previous study on specific phobias, we argued that trait anxiety could be one reason for reduced prefrontal inhibition [23, 24]. This would be consistent with studies indicating that people with anxiety disorders have higher trait anxiety scores than those who are not anxious [27]. It has additionally been described that trait anxiety scores are even higher in social phobias and GAD than in specific phobias [27, 28]. In the present study, we examined the correlation between trait anxiety scores and the N100 amplitudes. We found indeed that the higher the trait anxiety score, the smaller (more positive) the N100 amplitude. A connection between trait anxiety and the DLPFC is supported by the findings of Saviola et al. [53], who described that trait anxiety can be associated with frontal brain regions, while state anxiety was associated with activity in the precuneus cortex.

It should be noted that the correlation between N100 amplitude and trait anxiety was independent of the diagnosis effect of social phobia/GAD on trait anxiety and the three groups differed in trait anxiety when corrected for the influence of the N100. The regression equations for each group had similar slopes, arguing for an additive effect of anxiety scores on the N100 score. Such an additive model could be relevant in the context of the potential treatment of anxiety symptoms using repetitive TMS (rTMS). With rTMS, subjects receive repeated magnetic stimulation with a frequency of 1 Hz (slow-frequency rTMS with mainly inhibitory effects) or 10-20 Hz (high-frequency rTMS with mainly excitatory effects) over a period of approx. 10-20 minutes for several weeks. Recently, high-dose protocols with various rTMS sessions in one day have gained attention. Studies have shown that anxiety symptoms decreased in some patients treated with rTMS in prefrontal areas [54]. If this symptom improvement could be attributed to the alignment of GABAB levels by rTMS, our model could make an important contribution regarding future responders and nonresponders to rTMS treatment. Exploratively, it might be that the N100 reduction is not as pronounced in subjects who respond less to treatment with rTMS as in subjects who respond well. Because of the correlation of the N100 with trait anxiety scores, a previously assessed trait anxiety score could already provide preliminary indications of response or nonresponse to rTMS. This is supported by a study that has shown a reduction of anxiety bias effects and positive memory processing in young subclinical adults with high anxiety scores after rTMS of the DLPFC [55]. Furthermore, it has been described that especially individuals with high anxiety levels benefited from the rTMS of DLPFC [56].

However, these hypotheses should be considered hypothetical and exploratory due to the lack of available studies.

In the psychotherapeutic treatment of anxiety patients, a repeated assessment of the N100 through a single TMS session, as conducted in this study under resting conditions, could be utilized to monitor biological treatment effects. In terms of positive reinforcement, this could enhance patients' motivation for therapy, although this would involve considerable effort.

In future clinical trials, the N100 could be incorporated as a biological marker to assess the mechanisms of new treatments.

Moreover, we also found no significant N100 reduction in a working memory task in this study. Accordingly, there was no modulation of N100 by a low-load 1-back task. A modulation effect might only become apparent at tasks with a higher working memory load. Additionally, we did not find the effect of emotional processing with smaller N100 amplitudes in memorizing fearful compared with neutral facial expressions in the group of social phobia and GAD that we found for specific phobia. Studies have shown that fearful facial expressions elicit higher levels of state anxiety. State anxiety has been associated with a higher limbic response in this context [57]. Our results show a modulation of prefrontal inhibition when memorizing fearful facial expressions. This modulation could possibly be due to a nonspecific bottom-up increase in arousal in the limbic system during the processing of fearful facial expressions. However, we found this modulation only in subjects with specific phobia and not in the social phobia and GAD group. A meta-analysis by Kenwood et al. [58] has described that in specific phobia, there is hyperactivity in limbic areas, whereas in social phobia and GAD, there is rather deactivation in the same areas. In addition, differences were described between the activation patterns of pathological anxiety of the specific anxiety disorder and induced anxiety. Specific phobias had the greatest convergence with induced anxiety, whereas social phobias and GAD had the least convergence with induced anxiety. It was suggested that induced anxiety was a better model for some subtypes of pathological anxiety than for others [58, 59]. This could be a rationale for the modulation exclusively in the specific phobia group in response to fearful compared to neutral facial expressions.

However, another possibility that could have led to the lack of an effect in all groups is the low number of trials (20 per emotion). This needs to be investigated in future studies with an increased number of trials to gain certainty.

### 4.2. Limitations

The use of TMS without neuronavigation could be a limitation. TMS was aligned to the localization described by Rusjan et al., which has been used in previous TMS-EEG studies [49, 52, 60].

Also, TMS was used without sensory masking, which could contaminate the TMS potential with auditory and somatosensory evoked potentials [61, 62]. However, AEPs have previously been described in the literature with an N100-P180 complex. The auditory N100 emerges with a central topography. The topography of the here-investigated TMS-evoked N100 is expected to be lateralized at the site of stimulation [49]. Similarly, trigeminal SSEPs are described with a contralateral topography around centroparietal electrodes [24].

Furthermore, it remains to be noted that we included different anxiety disorder subtypes in the group of social phobia and GAD. Therefore, a systematic investigation of the subtypes of social phobia and generalized anxiety disorder separately should be the subject of future studies.

### 4.3. Conclusion

We were able to extend our previous findings of a reduced inhibition in the DLPFC in subjects with specific phobia [23, 24] to subjects with social phobia and GAD. Subjects diagnosed with an anxiety disorder showed impaired GABAB-mediated inhibition reflected in a smaller amplitude of the TMS-evoked N100.

Our main finding was the relationship between trait anxiety and the N100. With increasing trait anxiety scores, the N100 amplitude decreases, which is likely to be an additive model of trait anxiety and anxiety diagnoses for inhibitory deficits in the DLPFC. This finding could be important in the context of responders and nonresponders in the potential treatment of anxiety symptoms with rTMS.

## Figures and Tables

**Table 1:** Sample and TMS characteristics.

	Group		
	Social phobia and GAD (*N* = 20)	Specific phobia (*N* = 21)	Control group (*N* = 24)
Gender (*n*)	16 females, 4 males	20 females, 1 male	21 females, 3 males
Age (mean ± SD, in years)	21.8 ± 2.1	22.2 ± 2.1	22.1 ± 1.7
Handedness (*N*)	17 right, 3 left	19 right, 2 left	24 right
IQ (CFT 20-R, mean ± SD)	115 ± 11.5	113 ± 11.9	115 ± 11.9
Primary diagnoses	Social phobia (*N* = 17) GAD (*N* = 3)	Specific phobia (*N* = 21)	None
Comorbid diagnoses	Mild depressive episode (*N* = 2)	None	None
Trait anxiety score (mean ± SD)	63.2 ± 1.8	47.5 ± 2.3	45.5 ± 1.6
Resting motor threshold (mean ± SD in % of stimulator output)	55.6 ± 9.9	52.8 ± 9.1	52 ± 9.8

GAD = generalized anxiety disorder; SD = standard deviation.

## Data Availability

The original data used to support the findings of this study have not been made available because the ethics committee did not grant permission to share study data with third parties or to upload data in anonymized form.

## Supplementary Materials

Supplementary Materials Further information on N100 amplitudes and latencies for all conditions can be found in Table 1 of the supplementary material.
