# Supplementary material for: Young Adults with Anxiety Disorders Show Reduced Inhibition in the Dorsolateral Prefrontal Cortex at Higher Trait Anxiety Levels: A TMS-EEG Study
Source: Depress Anxiety. 2024 May 30;2024:2758522. doi: 10.1155/2024/2758522 (PMC11918925; doi:10.1155/2024/2758522)
Supplement: Supplementary Materials — Further information on N100 amplitudes and latencies for all conditions can be found in Table 1 of the supplementary material. [file 2758522.f1.docx]

**Supplementary material**

Table 1. *N100 amplitudes (*µV+SD) *and latencies (ms+SD) for all conditions.*

|  | | **Social phobia and GAD** | | **Specific phobia** | | **Control group** | |
| --- | --- | --- | --- | --- | --- | --- | --- |
| DLPFC at rest | |  | |  | |  | |
| N100 left latency ms±SD* amplitude µV±SD | | 110.6±15.6  -1.6±3.4 | | 113.6±16.6  −2.3 ± 3.7 | | 115.8±15.2  −3.8±4.3 | |
| N100 right latency ms±SD amplitude µV±SD | | 115.1±18.0  -3.4±3.6 | | 115.6±9.1  −3.3±3.1 | | 113.8±16.1  −5.5±4.1 | |
| DLPFC task latency ms±SD amplitude µV±SD | | 117.8±11.9  -2.9±3.1 | | 110.6±16.6  −3.8±3.1 | | 123.6±11.4  −6.4±3.7 | |
| Emotional facial expressions | |  | |  | |  | |
| Fearful latency ms±SD  amplitude µV±SD | | 117.5±14.2  -3.3±-3.6 | | 114.8±16.1  −1.9±3.7 | | 113.0±17.2  −5.6±3.7 | |
| Angry latency ms±SD  amplitude µV±SD | | 110.7±15.9  -1.9±6.9 | | 113.7±14.1  −3.7±7.6 | | 117.4±15.5  −6.0±4.5 | |
| Neutral latency ms±SD  amplitude µV±SD | | 117.5±14.2  -1.6±5.2 | | 111.1±14.8  −4.2±4.4 | | 118.9±15.5  −6.1±4.4 | |
|  | |  | |  | |  | |

*SD=standard deviation
